# Elevational characteristics of soil bacterial community and their responses to soil translocation at a mountainside in northwest Sichuan, China

**DOI:** 10.1038/s41598-023-44811-2

**Published:** 2023-10-20

**Authors:** Xuemei Wang, Tianzhi Huang, Yunyun Li, Guang Zhao, Jixia Zhao

**Affiliations:** 1 Ecological Security and Protection Key Laboratory of Sichuan Province, Mianyang Normal University, Mianyang, 621000 Sichuan China; 2 School of Resource and Environmental Engineering, Mianyang Normal University, Mianyang, 621000 Sichuan China; 3 Key Laboratory of Ecosystem Network Observation and Modeling, Institute of Geographic Sciences and Natural Resources Research, Chinese Academy of Sciences, Beijing, 100101 China; 4 College of Resources and Environment, Yunnan Agricultural University, No. 95, Heijin Road, Panlong District, Kunming, 650201 China

**Keywords:** Ecology, Climate sciences, Environmental sciences

## Abstract

How the soil bacterial communities vary with elevation is context-dependent, and the effect of soil translocation between elevations on bacterial community structure and metabolic function was not fully understood yet. Here, the bacterial community composition and diversity at five elevations along a 1600–3000 m elevation gradient on a mountainside in northwest Sichuan were characterized, and the responses of soil bacterial community to simulated climate changes were further studied by soil translocation reciprocally at three elevations for 12 months. Significant differences were found in soil temperature and moisture at different elevations, but there was no observed change in bacterial alpha diversity. The relative abundance of bacterial phyla was significantly different among the five elevations except for Proteobacteria (the dominant bacterial phyla in five elevation), and most bacterial phyla correlated with soil temperature, moisture, pH and soil bulk density. The direct effect of soil properties (pH, soil nutrients and soil bulk density) on soil bacterial community was stronger than the direct effect of temperature and moisture. Soil translocation changed the relative abundance of some bacterial phyla, and taxonomic groups with significant changes were mainly non-dominant phyla rather than the dominant phyla. Metabolism was the primary function of bacterial community at all elevations, which accounted for ~ 80% of relative abundance, and soil translocation had little effect on metabolic function. These findings indicated that soil bacterial dominant taxa and soil bacterial metabolic functions are relatively stable, which contribute to the stability of the ecosystem when response to the climate change in the future.

## Introduction

The great diversity of soil microorganisms living underground has significantly promoted the formation of aboveground biodiversity and the function of terrestrial ecosystems^1^. Bacteria are a large group of microorganisms in soil, which contribute to organic matter decomposition, mineral weathering, nutrient cycling, and other important soil processes^2^. The soil bacterial community structure and their functions are sensitive to environmental changes, and they are affected by a variety of environmental factors^3^. Among the many influencing factors, climate change, including temperature and moisture regimes, is one of the important factors affecting bacterial communities^4^. Understanding the responses of soil bacterial communities to climate change has aroused widespread interest among ecologists^5,6^.

Mountains are one of the components of terrestrial systems and have important ecosystem functions^7^. Gradients in temperature and soil moisture along elevations of a mountain provide a natural laboratory for exploring their influence on bacterial distributions^8,9^. Many studies have shown that bacterial community diversity has a clear spatial distribution pattern in the elevation gradient, such as a diversity decline^3,7,10^, a ‘peak’ in mid-elevation^11–13^, or no trend with increasing elevation^14–16^. The soil bacterial community distribution along elevational gradient was affected by the temperature and moisture, soil chemical properties, and vegetation^9,17^. Therefore, the elevation gradient can explain the responses of soil bacterial communities to climate change in some degree.

Artificial translocation of soil or soil–plant systems across different regions along an elevational gradient can change the temperature and moisture conditions, which is an additional method for simulating climate change^6,18,19^. Some studies have shown that soil bacterial communities are significantly affected by short-term (13 months and 2 years) soil translocation^18,20,21^. The average level of adaptation was 77% after 2 years, and complete after 11 years^22^. However, soil microbial communities might have a high resistance to the experimental climate manipulation, and thus, studies also have shown that the short-term soil translocation (2 year)^19^, medium term (4 years) soil translocation^23^, and even a long-term (17 years) reciprocal soil translocation have revealed no significant effects on microbial community structures^24^. Therefore, the response of soil bacterial communities to soil translocation requires further study.

The mountainside in northwest Sichuan, located between the Tibetan Plateau and Sichuan Basin, is an important part of the ecological barrier of the upper reaches of the Yangtze River. Under the background of global warming, the Tibetan Plateau is highly sensitive to global climate change^12,13,20^, and the climate of western Sichuan has also been affected^25^. In this study, the characteristics of soil bacterial community structure along an elevation gradient on a mountainside in northwest Sichuan were explored using high-throughput sequencing technology, and the responses of soil bacterial communities to climate change were further studied by reciprocal soil translocation at three elevations with 1000 m interval. In addition, the function of soil microorganisms is closely related to microbial community composition^26,27^, and Phylogenetic Investigation of Communities by Reconstruction of Unobserved States (PICRUSt) analysis can reliably predict the metabolic functions of soil bacterial communities^28^. Therefore, the PICRUSt method was used to predict bacterial community functions according to 16S rRNA sequencing data. It was hypothesized that: (1) alpha diversity of soil bacterial would first decrease and then increase due to the decreased temperature and increased moisture along the elevation gradient, and different bacterial phyla show diverse trends with the increasing elevation; (2) soil bacterial community composition would respond significantly to soil translocation due to the changes of temperature and moisture; and (3) changes in community composition would drive changes in metabolic functions.

## Materials and methods

### Study sites

The study site was located in Huya Township, Pingwu County in the northwest Sichuan, where belongs to the Xuebaoding National Nature Reserve, which is the main peak of Minshan Mountain. Pingwu County (31°59′31″–33°02′41″N, 103°50′31″–104°59′13″E) is situated in the mountainous areas in transition from Sichuan basin to Qinghai Tibet Plateau in the southwest, and it is at the upper reaches of Fujiang River, a tributary of the Yangtze River. Pingwu County covers an area of 5974 km^2^, where the highest elevation is 5400 m, and the lowest is 600 m. The mountainous areas above 1000 m account for 94.33% of the total area. Because of the great differences in elevation, the climate changes vertically with elevation. The annual average temperature is 14.7 °C, and the annual average precipitation is 839.9 mm.

### Experimental setup and soil sampling

#### Soil sampling at different elevations

**Figure 1:**
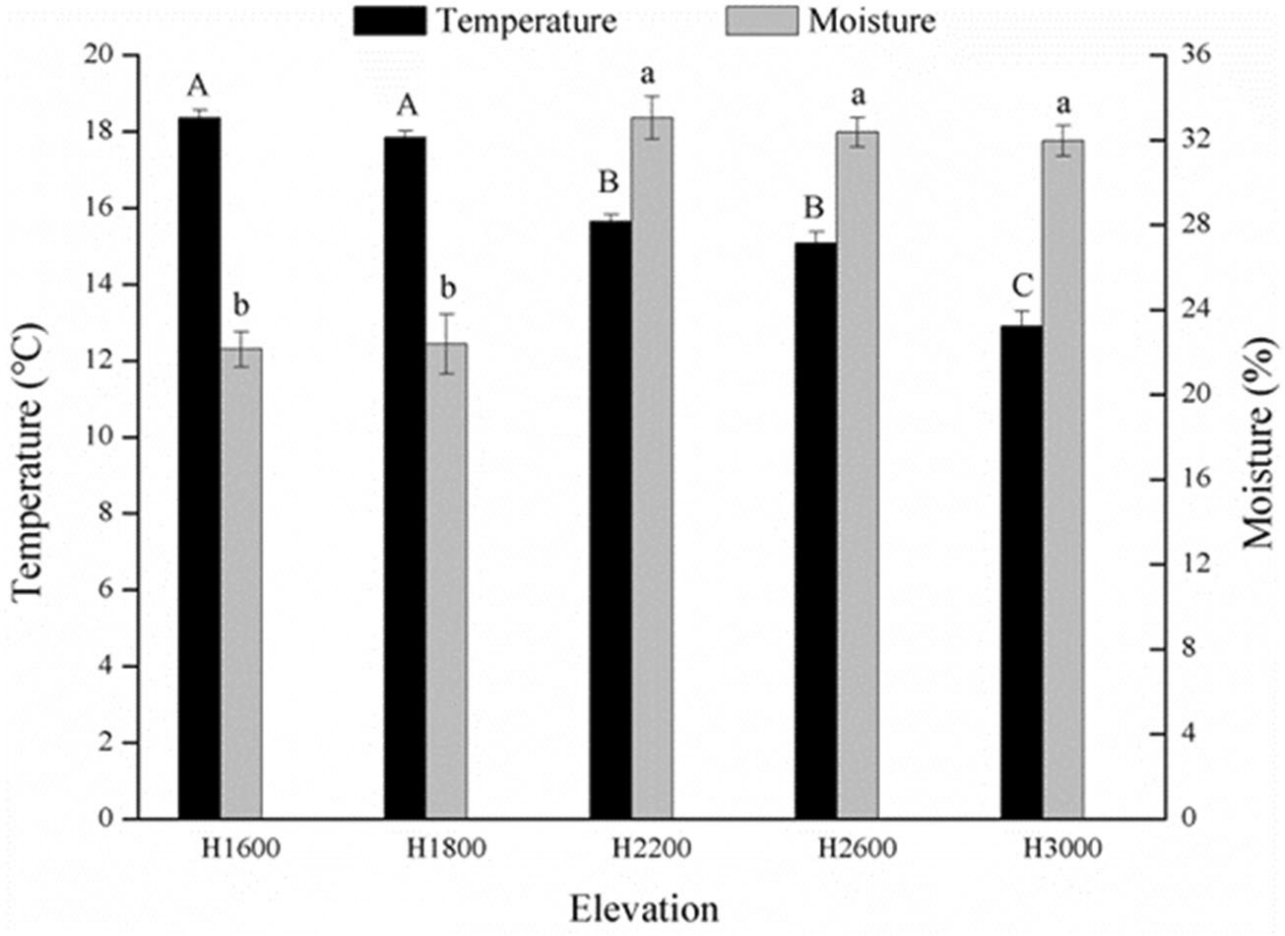
Soil temperature and moisture from 1600 to 3000 m. Different capital letters above the bar indicate significant difference of temperature among different elevations, and different lowercase letters indicate significant difference of moisture among different elevations (*P* < 0.05).

Soil sampling was conducted from five sites with different elevations from 1600–3000 m in Xuebaoding Huya Nature Reserve in July 2019. The five sites were labeled as H1600, H1800, H2200, H2600, and H3000 with GPS used to record the longitude, latitude, and elevation of the sampling sites, and the vegetation and soil type of each site were investigated (Table 1). At each elevational site, we defined three plots (2 m × 2 m) located about 20–30 m apart as three independent replicates. In each plot, 0–20 cm soil samples were collected by sampling in five locations and composited together. Besides, a cutting ring was used for sampling the undisturbed soil in each plot and then testing the soil bulk density (SBD). During sampling, soil moisture and temperature at 20 cm depth were recorded with a soil thermo-hygrometer (TR-6, Shunkeda Co., Ltd., Beijing, China) (Fig. 1). Soil composited samples were placed in sterile polyethylene bags and transported to the laboratory by an icebox. Then, they were sieved with a 2 mm mesh to remove visible grass roots and stones. A part of the soil was used to measure the SBD, pH, soil organic matter (SOM), total nitrogen (TN), total phosphorus (TP), total potassium (TK), available nitrogen (AN), available phosphorus (AP), and available potassium (AK) (Table S1) according to the standard methods recommended by the Chinese Society of Soil Science^29^, and another part of the soil was stored at − 80 °C for genomic DNA extraction.

**Table 1:** Environmental information of the soil sampling sites in five elevations.

Sites	Elevation (m)	Longitude	Latitude	Main vegetation	Soil type
H1600	1627.19	104°03′20″	32°31′43″	*Alnus cremastogyne, Tetradium ruticarpum, Pteris multifida, Duchesnea indica, Boehmeria spicata, Artemisia carvifolia*	Mountain yellow–brown soil
H1800	1844.48	104°03′22″	32°32′02″	*Magnolia officinalis, Avena fatua, Kolkwitzia amabilis, Anthriscus sylvestris, Cotoneaster horizontalis, Aralia chinensis, Cedrus deodara*	Mountain yellow–brown soil
H2200	2252.85	104°03′12″	32°32′36″	*Phlomis umbrosa, Vitis amurensis, Spiraea salicifolia, Astilbe chinensis, Cyclosorus interruptus*	Mountain brown soil
H2600	2653.38	104°01′52″	32°32′53″	*Cyclosorus interruptus, Rhododendron calophytum, Ligularia sibirica, Meconopsis chelidonifolia*	Mountain brown soil
H3000	3013.78	104°00′59″	32°33′03″	*Hydrocotyle sibthorpioides, Hypolepis punctata, Parathelypteris glanduligera, Thuidium cymbifolium, Indocalamus tessellatus*	Mountain dark-brown soil

#### Soil translocation experiment

The responses of bacterial communities to climate change were further revealed by conducting a reciprocal translocation of soil cores at three sites, H1600, H2200, and H2600 with 1000 m interval. Intact soil cores (10 cm diameter × 20 cm depth) containing undisturbed soil and fine roots were translocated. In July 2019, nine intact mineral soil cores were excavated at each site, and the soil cores were placed in 150 μm nylon mesh bags. Three of these soil bags were left in situ at the site and the other six soil bags were transferred to the other two sites (downslope = ‘warmed’, upslope = ‘cooled’)^6^. Three plots of 1 × 1 m were set at each site, and each plot filled with the situ and translocated soil bags. For H1600, each plot was filled with a situ soil bag (H1600) and two translocated soil bags (H2200–1600, the down-moved soil bag from 2200 to 1600 m, and H2600–1600, the down-moved soil bag from 2600 to 1600 m). Similarly, for H2200, each plot was filled with a situ soil bag (H2200) and two translocated soil bags (H1600–2200, the up-moved soil bag from 1600 to 2200 m, and H2600–2200, the down-moved soil bag from 2600 to 2200 m). The situ soil bag and two translocated soil bags for H2600 were recorded as H2600, H1600–2600 and H2200–2600, respectively. Therefore, there were three situ soil bags and six translocated soil bags in each site, resulting in 27 soil bags in total. All bags were uniformly placed in 10–20 cm depth of each plot.

After 12 months of incubation in its situ or translocated sites, the bags were removed from the plots and transported to the laboratory using an icebox in July 2020. All samples were sieved with a 2 mm mesh and stored at − 80 °C until further processing.

### Soil bacterial DNA extraction, amplicon library preparation, and sequencing

About 0.5 g fresh soil was weighed from each sample, and total DNA was extracted using the Mobio Powersoil DNA Isolation kit (Mobio Laboratories, Carlsbad, CA, USA) according to manufacturer’s instructions. Extracted soil DNA was quantified using a NanoDrop ND-2000c UV–Vis spectrophotometer (NanoDrop Technologies, Wilmington, DE, USA), and the quality of DNA was detected using 1.2% agarose gel electrophoresis.

A paired-end dual-index sequencing approach was used. Bacterial 16S rRNA genes (v3-v4) were amplified by PCR using the primers 338F (5′-ACTCCTACGGGAGGCAGCA-3′) and 806R (5′-GGACTACHVGGGTWTCTAAT-3′)^30^. The amplification procedure was as follows: 95 °C for 2 min; 25 cycles at 95 °C for 30 s, 55 °C for 30 s, 72 °C for 30 s; and a final extension at 72 °C for 5 min. Three replicate PCRs were carried out for each DNA sample and subsequent products were pooled together. The PCR products were confirmed using agarose gel electrophoresis and subsequently isolated from the gel and purified using a Gene JET gel extraction kit (Thermo Fisher Scientific, Waltham, MA, USA).

The standard Illumina Truseq DNA library preparation process was used to construct the required genomic library. The library was quantified on Promega QuantiFluor using Quant-iT PicoGreen dsDNA Assay Kit (Invitrogen Corp., Carlsbad, CA, USA). After the library was qualified, purified PCR products were sequenced using an Illumina MiSeq platform (Illumina Inc., San Diego, CA) at Shanghai Personal Biotechnology Co., Ltd. (Shanghai, China).

### Bioinformatics analysis

Bacterial sequences were processed with the Quantitative Insights Into Microbial Ecology (QIIME) software package. Reads containing ambiguous bases were discarded; only overlapping sequences longer than 10 bp were assembled. After sequence optimization and data quality control, operational taxonomic units (OTUs) were clustered with ≥ 97% high-quality sequences similarity and OTU representative sequences were annotated. To eliminate the bias on diversity comparison caused by unbalanced sequencing, each sample was resampled at a depth of the minimum sequence of the sample detected. The RDP classifier method was used to compare OTU sequences of bacteria with that of Silva Database (http://www.arb-silva.de)^31^. The observed species (OTUs) and Shannon index were calculated to compare alpha diversities.

Furthermore, bacterial community functions at different elevations were predicted by PICRUSt2^28^. The Kyoto Encyclopedia of Genes and Genomes (KEGG) database was used to identify the functional genes^32^, and functional predictions were produced from the KEGG database using 16S rRNA data.

### Statistical analysis

One-way ANOVA was applied to analyze the effects of elevation on measured data, and differences among the five elevations were compared using Tukey’s Honestly Significant Difference (HSD) Test. Normality of data was tested using the Shapiro–Wilk test before analysis and data were normalized if necessary. Correlation analysis was conducted to reflect the relationships between the relative abundance of bacterial phyla and environmental factors, and between soil bacterial alpha diversity and environmental factors. All these analyses were conducted using SPSS25.0 for Windows.

Also, analysis of similarities (ANOSIM) were performed based on the Bray–Curtis dissimilarity matrix to identify whether there were significant differences among different elevations, and non-metric multidimensional scaling (NMDS) analysis based on the relative abundance of soil bacterial phyla was performed to visualize differences of the bacterial communities among five elevations using the ‘Vegan’ package in R. Additionally, the environmental factors were fitted into the NMDS analysis to reflect the correlations between bacterial community compositions and environmental factors. Furthermore, partial least squares structural equation modeling (PLS-SEM) was conducted to test for possible causal relationships between elevation, environmental factors, and soil bacterial community by employing SmartPLS3 software with a PLS algorithm. The preliminary theoretical basis of the model was that soil temperature and moisture were obviously different along the elevation gradient, and the changes of temperature and moisture would directly affect soil physical and chemical properties, which also affected soil bacterial community, so soil temperature and moisture would directly and indirectly affect soil bacterial community. The standardized root mean square residual (SRMR) and normed fit index (NFI) were used to evaluate the structural model^33,34^.

The effects of translocation on relative abundance of dominant bacterial phyla and predicted functions were analyzed by calculating the ratios of the relative abundance in situ and in translocated treatments^21^, and the statistical differences between the ratio and 1 were quantified by one sample T-test. Besides, significant differences in taxa (5 taxonomic levels from phylum to genus) among in situ and in translocated soils were further analyzed using linear discriminant analysis (LDA) effect size (LEfSe) analysis (http://huttenhower.sph.harvard.edu/lefse/), and the threshold on the logarithmic score of LDA analysis was set to 2.0^35^.

## Results

### Soil bacterial community composition and diversity in different elevations

Amplicon sequencing resulted in approximately 4,289,159 total sequences, averaging approximately 130,000 per sample. The depth of the minimum high quality sequences of these samples was 70,000 approximately, and thus, resampling was employed at a depth of 70,000 sequences per sample. All the rarefaction curves of the samples were asymptotic lines, suggesting that the sequencing results could basically reflect the real situation of these samples; and the species accumulation were in the range of 2000–5000 (Fig. S1). The number of OTUs detected in samples increased from 2781 to 5215, with an average of 4243, and the bacterial Shannon index was 10.23 to 11.07. Both the OTUs and Shannon index were not significant different among five elevations (*P* > 0.05) (Fig. S2), and they were not significantly correlated with soil temperature, moisture and other soil physical and chemical properties (*P* > 0.05) (Table S2).

**Figure 2:**
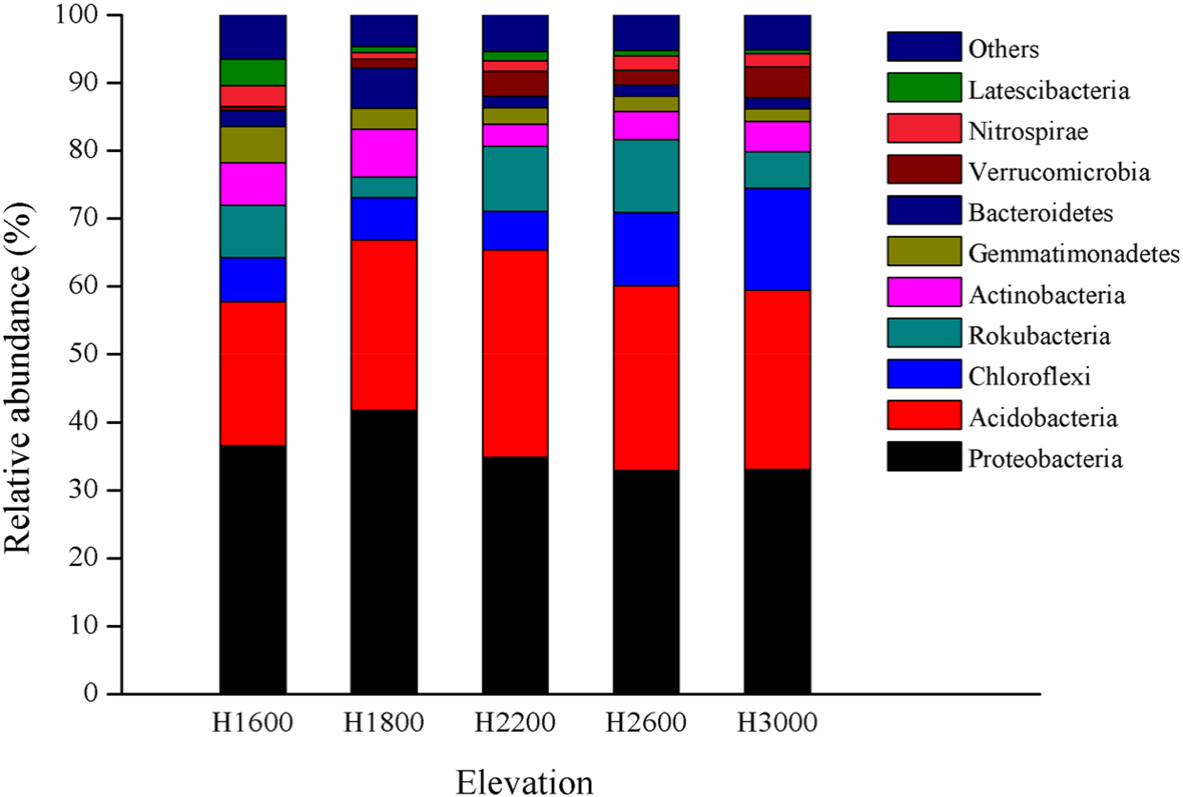
Bacterial phyla composition and relative abundance in soils under five elevations.

A total of 33 phyla, 92 classes, 191 orders, 272 families, and 370 genera were detected in all samples. The top 10 phyla were Proteobacteria, Acidobacteria, Chloroflexi, Rokubacteria, Actinobacteria, Gemmatimonadetes, Bacteroidetes, Verrucomicrobia, Nitrospirae, and Latescibacteria, accounting for > 94% of the total bacterial sequences, and the others only accounted for ~ 5%. The dominant bacterial phyla in the five elevations were Proteobacteria and Acidobacteria, accounting for 53.55–67.52%, with an average of 61.96%. The top 10 phyla differed among different elevations (*P* < 0.05), except for Proteobacteria (*P* = 0.052) (Fig. 2; Table S3).

**Figure 3:**
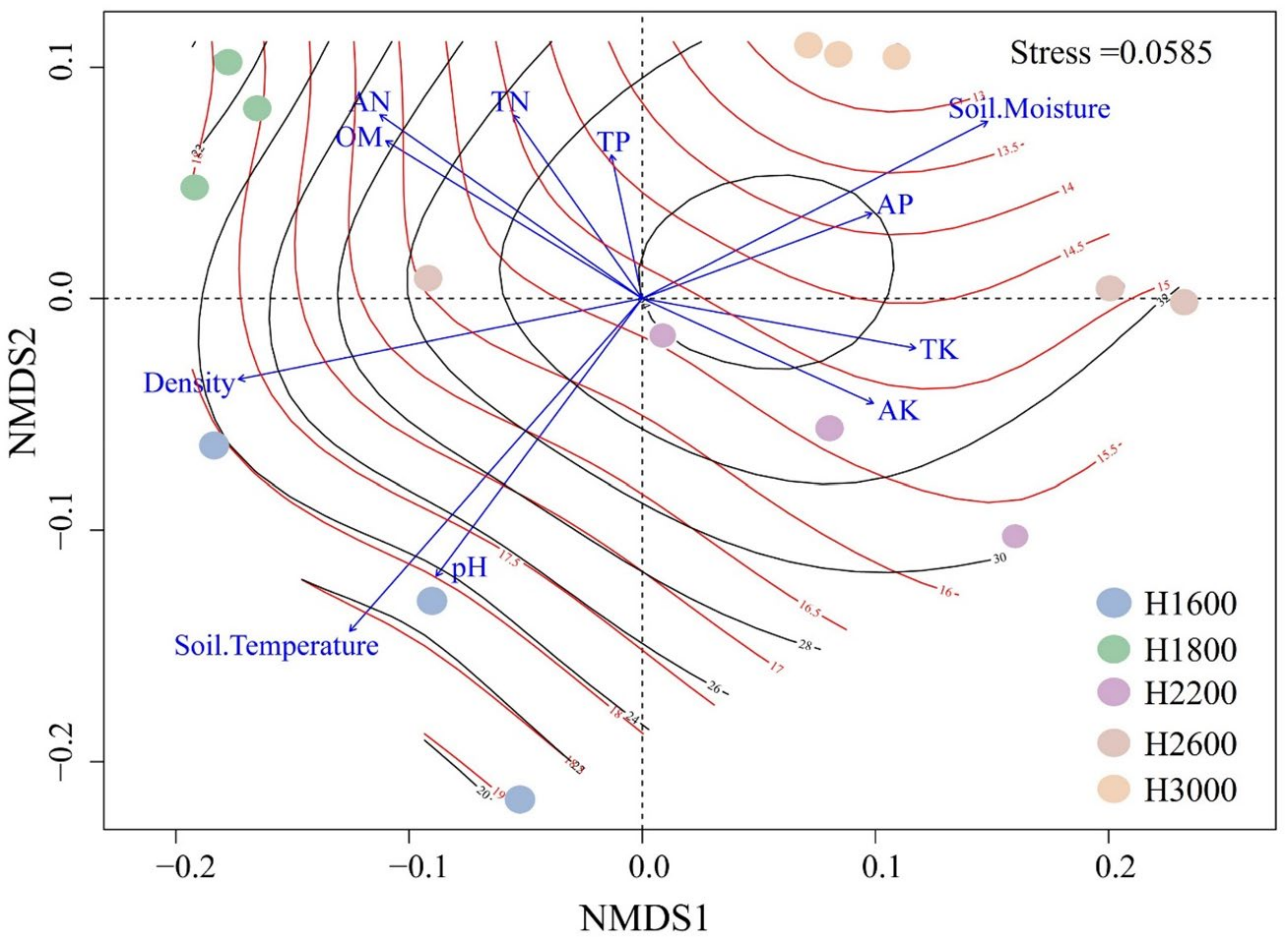
NMDS results for bacterial communities in five elevations and the fitting results of environmental factors. Contour lines represent the surface fitting results of environmental factors to NMDS1. Black contours represent moisture, and the red contours represent temperature.

Although elevation did not alter the alpha diversity of the bacterial community, it altered the beta diversity of the bacterial community. ANOSIM analysis revealed that elevation (r = 0.930, *P* = 0.001) significantly affected bacterial community structure. NMDS analysis based on bacteria phylum level showed that samples from H1600 and H1800 clustered on the left side of the first axis, while samples from H2200, H2600 and H3000 clustered on the right side (Fig. 3). Among them, H1600 and H1800 had 1000 common OTUs, while H1600 only had 175, 182 and 108 common OTUs with H2200, H2600 and H3000, respectively. The number of common OTUs of H2200, H2600 and H3000 was 706, and 44 for the five elevations (Fig. S3).

### Relationships between soil bacterial community composition and the soil variables

**Figure 4:**
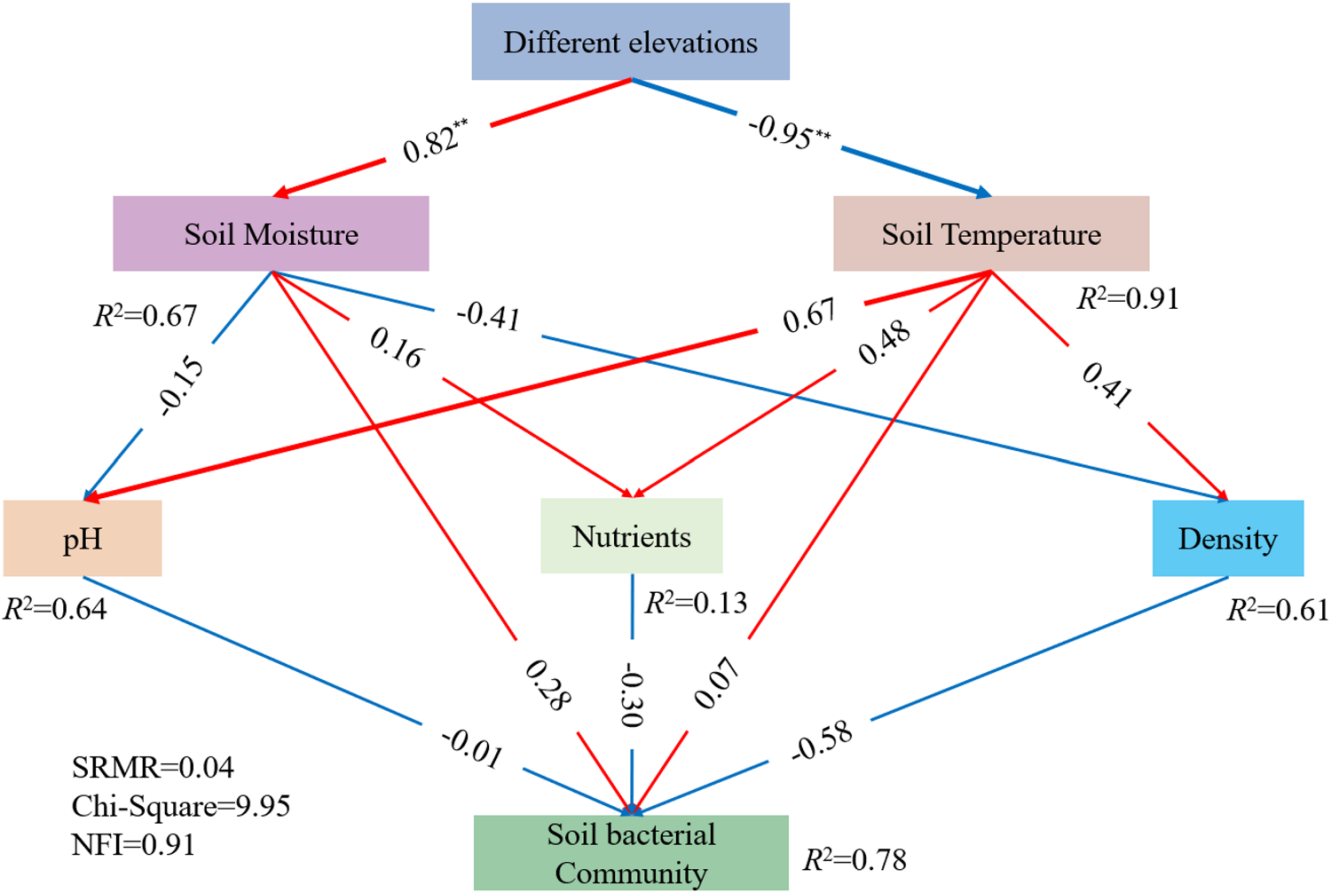
Partial least squares structural equation model (PLS-SEM) visualizing and calculating the relationships between soil bacterial community and its affecting factors. *SRMR* standardized root mean square residual, *NFI* normed fit index. Red arrows indicate positive effects, and blue arrows indicate negative effects, and the thickness of the arrow line indicates the magnitude of the path coefficient in the model. The numbers on arrows are standardized path coefficients.

From the NMDS analysis, soil temperature, SBD, soil moisture and pH were the significant factors affecting soil bacterial community. Besides, SOM and AN also affected soil bacterial community (Fig. 3; Table S4). Most bacterial phyla were correlated with soil temperature, SBD, soil moisture and pH (Table S5), and these parameters were therefore selected into the PLS-SEM model. In addition, some of the phyla were related to SOM, AN, TN, TK, AP and AK (Table S5), and all these parameters represented soil nutrients. Therefore, we applied principal component analysis (PCA) to the nutrient parameters (SOM, TN, TP, TK, AN, AP, AK) and incorporated the first axis of PCA results (explained 51.0% of the total variance) into the PLS-SEM model. The PLS-SEM showed an adequate model fit with SRMR < 0.08 and NFI > 0.9 (Fig. 4). The PLS-SEM results showed that elevation had a direct and positive effect on soil moisture [standardized path coefficients (pc) = 0.82], whereas elevation exhibited direct negative effects on soil temperature (pc = − 0.95). Then soil temperature (pc = 0.07) and soil moisture (pc = 0.26) directly influenced soil bacterial community. Additionally, soil temperature directly and positively influenced soil pH (pc = 0.67), soil nutrients (0.48) and SBD (pc = 0.41), while soil moisture directly and negatively influenced soil pH (pc = − 0.15) and SBD (pc = − 0.41), and positively influenced soil nutrients (0.16). And finally, the soil pH (pc = − 0.01), soil nutrients (− 0.30) and SBD (pc = − 0.58) directly altered soil bacterial community. Therefore, the indirect effect of soil moisture and temperature on soil bacterial community was mediated by soil pH, soil nutrients and SBD. Detailed standardized effects of these factors were provided in Table S6.

### Changes of soil bacterial community compositions after soil translocation

**Figure 5:**
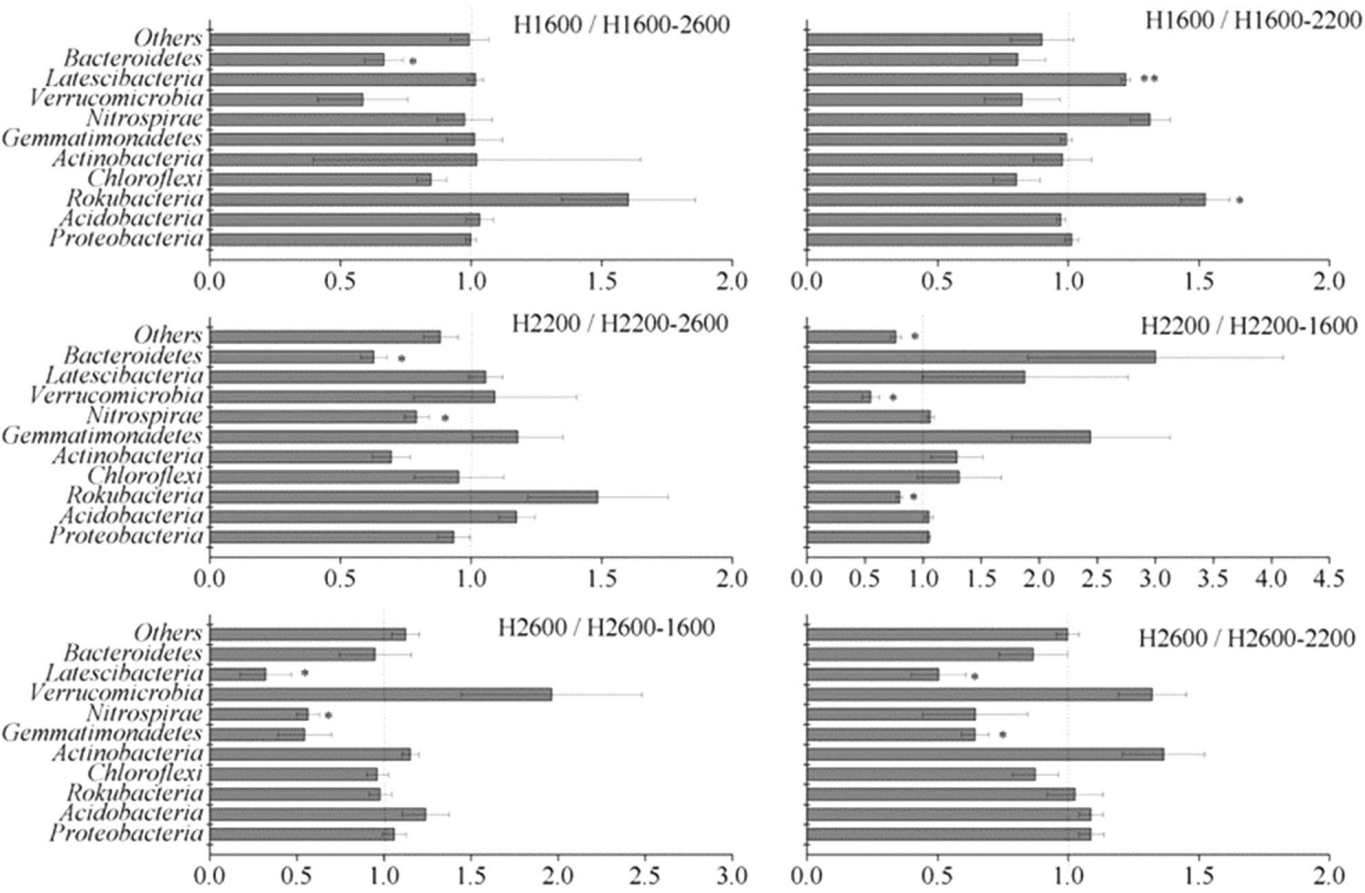
The ratios of relative abundance of soil bacterial phyla in situ and translocated treatments. * and ** showed that there were significant differences between situ and translocated treatments at the level of 0.05 and 0.01, respectively, and the same as in Fig. 7.

**Figure 6:**
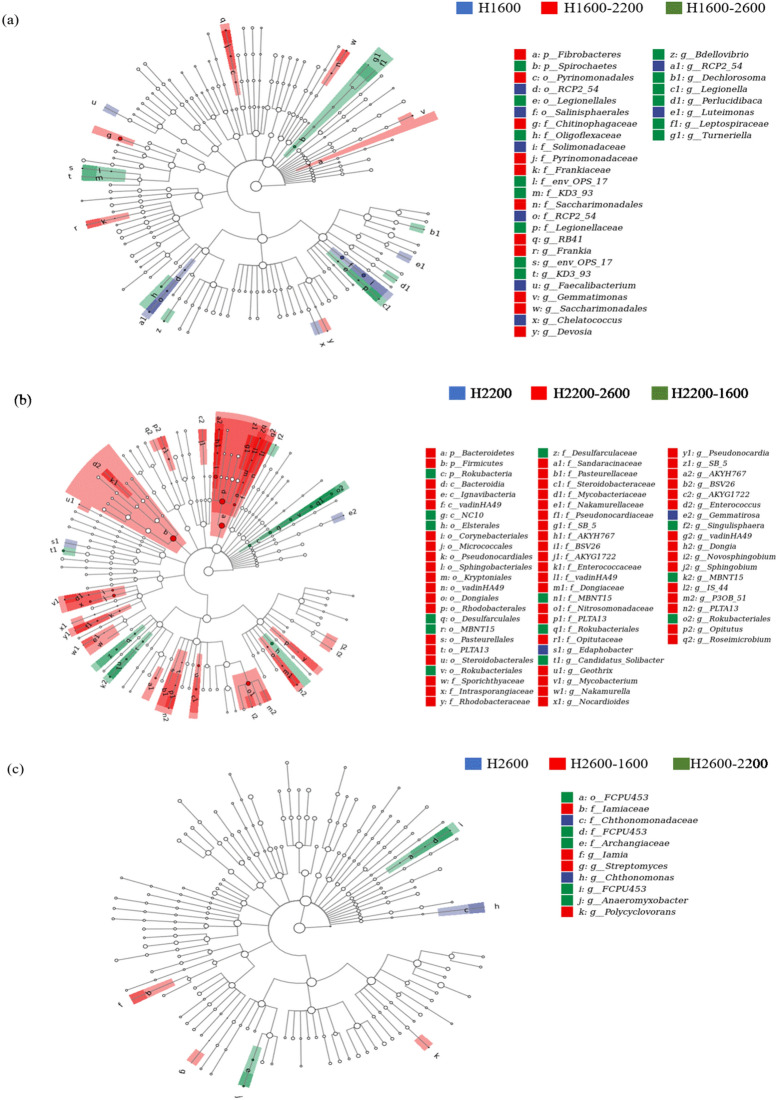
LEfSe analysis showing soil bacterial community differences between the situ and translocated treatments. The five circulars in the figure represent the phylogenetic level of phyla, class, order, family and genus from the inside out. Taxa (biomarkers) with significant higher relative abundance in different treatments were colored.

Analysis of bacterial community compositions at the phylum level showed that Proteobacteria and Acidobacteria were still dominant after soil translocation, and soil translocation had no significant effect on their relative abundance (Fig. 5). The relative abundance of Rokubacteria was significantly decreased after translocation to higher elevation (H1600–2200) and significantly increased after translocation to lower elevation (H2200–1600). Similarly, the relative abundance of Gemmatimonadetes (H2600–2200), Verrucomicrobia (H2200–1600), and Latescibacteria (H2600–2200, H2600–1600) tended to be increased after translocation from higher elevation to lower elevation, and the relative abundance of Latescibacteria decreased when translocated from lower to higher elevation (H1600–2200). However, the relative abundance of Bacteroidetes was increased after soil was translocated from lower (H1600, H2200) to higher elevation (H2600). Soil translocation did not significantly change the relative abundance of Chloroflexi and Actinobacteria. The ratios of the abundance in situ and in translocated treatments were in the range of 0.32–3.00. LEfSe analysis showed (Fig. 6) that 33 biomarkers were sensitive to soil translocation after low elevation soil (H1600) translocated to higher elevations (H2200; H2600) (*P* < 0.05, LDA > 2.0), and 11 groups showed significant differences after H2600 soil translocated to lower elevations (H2200; H1600), and more groups changed significantly after H2200 soil translocated. Among the taxonomic groups with significant differences, the groups belonged to the Proteobacteria and Acidobacteria only accounted for 39.5%.

### Metabolic function prediction of soil bacterial community

Bacterial community functions at different elevations were predicted by PICRUSt based on KEGG database. Six primary metabolic pathways (pathway level 1) were obtained at the five elevations. The highest relative abundance was metabolism, with ~ 80% at all elevations, and then in sequence were genetic information processing, cellular processes, and environmental information processing (Table 2). The relative abundance of organismal systems and human diseases was lower than 1%. The relative abundance of metabolism was highest at H1800, but H1800 showed the lowest relative abundance of genetic information processing. In addition, the relative abundance of cell processes at H1600 and H1800 were significantly lower than those at the other three elevations. A total of 34 secondary metabolic pathways (pathway level 2) were obtained, including 17 with relative abundance > 1%. The five most dominant of secondary metabolic pathways consisted of amino acid metabolism, carbohydrate metabolism, metabolism of cofactors and vitamins, metabolism of terpenoids and polyketides, and metabolism of other amino acids, and they were significantly different at different elevations.

**Table 2:** Relative abundance of functional metabolic pathways in soil bacterial community at different elevations. Different lowercase letters indicate significant difference among different elevations (*P* < 0.05).

Pathway level 1	Pathway level 2	H1600	H1800	H2200	H2600	H3000
Cellular Processes	Cell growth and death	1.486 ± 0.006 a	1.447 ± 0.002 a	1.466 ± 0.003 a	1.490 ± 0.016 a	1.478 ± 0.009 a
	Cell motility	2.517 ± 0.029 b	2.392 ± 0.022 b	3.013 ± 0.035 a	3.008 ± 0.135 a	2.905 ± 0.027 a
	Total	4.003 ± 0.028 b	3.840 ± 0.024 b	4.479 ± 0.034 a	4.498 ± 0.151 a	4.384 ± 0.036 a
Environmental Information Processing	Membrane transport	1.571 ± 0.008 a	1.594 ± 0.004 a	1.581 ± 0.018 a	1.587 ± 0.021 a	1.614 ± 0.008 a
Genetic Information Processing	Folding, sorting and degradation	3.537 ± 0.026 a	3.239 ± 0.019 b	3.395 ± 0.009 ab	3.514 ± 0.082 a	3.387 ± 0.024 ab
	Replication and repair	5.158 ± 0.022 a	4.880 ± 0.005 b	5.022 ± 0.026 ab	5.152 ± 0.094 a	5.074 ± 0.031 ab
	Translation	3.106 ± 0.022 a	2.796 ± 0.017 b	2.899 ± 0.014 ab	3.035 ± 0.100 a	2.920 ± 0.025 ab
	Total	11.803 ± 0.071 a	10.916 ± 0.042 b	11.317 ± 0.049 ab	11.701 ± 0.277 a	11.382 ± 0.081 ab
Metabolism	Amino acid metabolism	12.811 ± 0.021 ab	12.859 ± 0.028 a	12.703 ± 0.029 ab	12.674 ± 0.032 b	12.692 ± 0.065 ab
	Biosynthesis of other secondary metabolites	2.950 ± 0.017 a	2.832 ± 0.100 ab	2.539 ± 0.101 b	2.526 ± 0.051 b	2.629 ± 0.079 ab
	Carbohydrate metabolism	12.676 ± 0.035 b	12.67 ± 0.010 b	12.844 ± 0.036 ab	12.697 ± 0.069 ab	12.904 ± 0.057 a
	Energy metabolism	5.292 ± 0.010 ab	5.188 ± 0.009 b	5.263 ± 0.014 ab	5.344 ± 0.058 a	5.306 ± 0.031 ab
	Glycan biosynthesis and metabolism	3.294 ± 0.012 a	3.150 ± 0.002 a	3.232 ± 0.039 a	3.257 ± 0.101 a	3.200 ± 0.022 a
	Lipid metabolism	6.234 ± 0.054 a	6.768 ± 0.017 a	6.530 ± 0.079 a	6.237 ± 0.255 a	6.564 ± 0.030 a
	Metabolism of cofactors and vitamins	12.582 ± 0.049 a	11.912 ± 0.042 b	12.162 ± 0.018 ab	12.550 ± 0.237 a	12.206 ± 0.088 ab
	Metabolism of other amino acids	7.814 ± 0.011 a	7.911 ± 0.008 a	7.872 ± 0.062 a	7.851 ± 0.062 a	7.845 ± 0.052 a
	Metabolism of terpenoids and polyketides	10.113 ± 0.045 a	9.813 ± 0.027 a	9.676 ± 0.272 a	9.929 ± 0.07 a	9.573 ± 0.388 a
	Nucleotide metabolism	1.660 ± 0.005 a	1.574 ± 0.003 a	1.584 ± 0.005 a	1.646 ± 0.044 a	1.591 ± 0.009 a
	Xenobiotics biodegradation and metabolism	4.782 ± 0.190 b	6.513 ± 0.168 a	5.903 ± 0.273 ab	5.193 ± 0.496 b	5.853 ± 0.077 ab
	Total	80.214 ± 0.135 b	81.195 ± 0.043 a	80.312 ± 0.064 ab	79.909 ± 0.417 b	80.368 ± 0.122 ab

**Figure 7:**
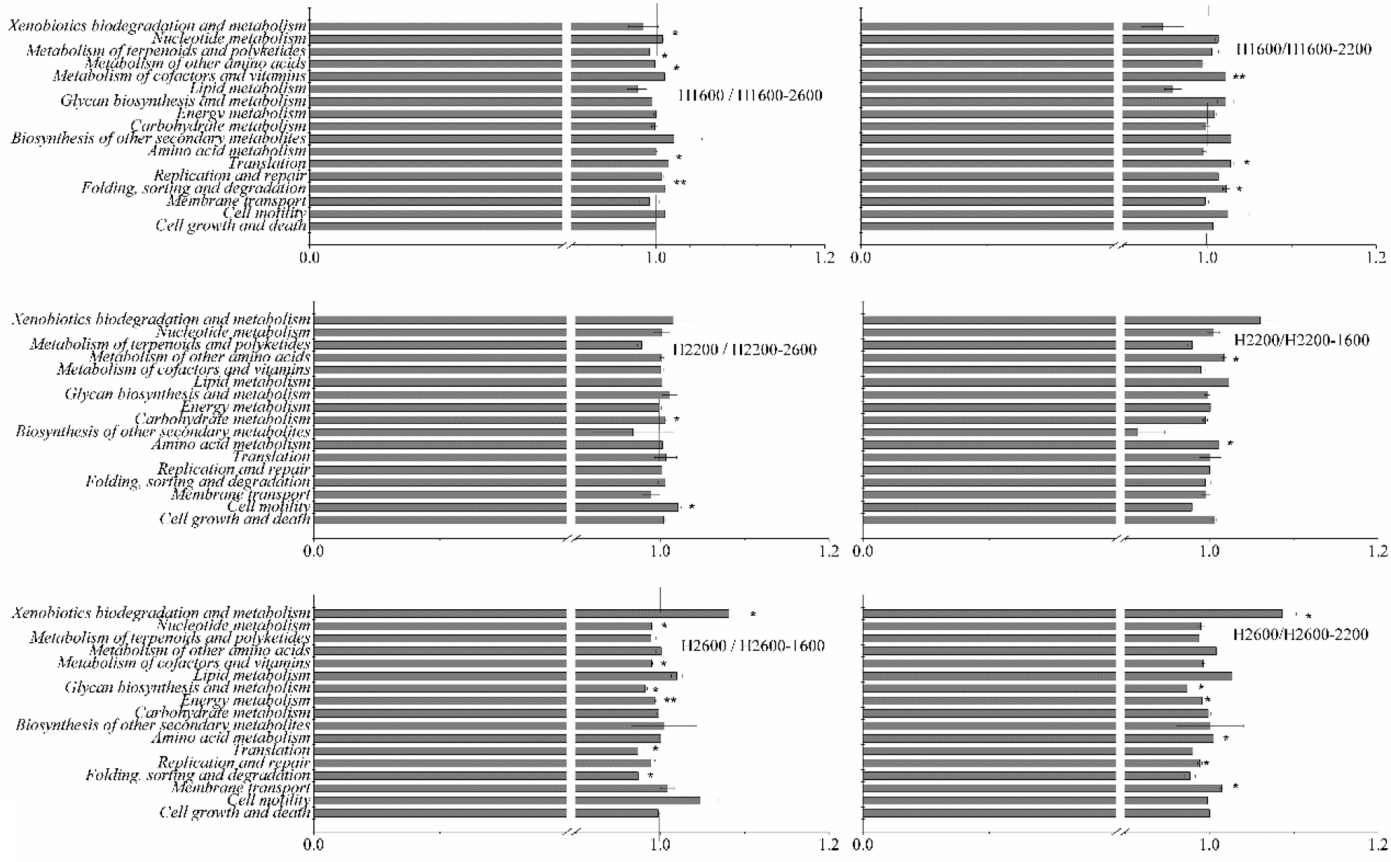
The ratios of relative abundance of functional metabolic pathways level 2 in situ and translocated treatments.

After soil translocation for a year at H1600, H2200, and H2600, bacterial functions clustered into metabolism were still dominant, and the metabolic functions of bacterial communities did not change significantly with soil translocation. Although some secondary metabolic pathways were changed by soil translocation, the ratios of the abundance in situ and in translocated treatments were relatively stable, with the range of 0.92–1.09 (Fig. 7).

## Discussion

### Effects of elevation on soil bacterial communities

The present results showed that no significant differences in soil bacterial alpha diversity were found at different elevations (Fig. S2), and OTUs and Shannon index were not correlated with environmental factors including soil temperature and moisture (Table S2), which was inconsistent with hypothesis 1. There have been many recent studies focus on the changes of soil bacterial composition and diversity along elevations in mountainous regions, and no consistent trend was found in soil bacterial diversity^7,12,25^. The inconsistent results of the trend of bacterial diversity with elevation might be explained by the following reasons. First, it might be due to the different elevation scales, that some studies have addressed bacterial diversity in small scale elevation gradients with a consistent ecosystem, while others have focused on large scale elevation gradients with large elevation intervals and ecosystem differences^8,36^. Li et al.^37^ found distinct soil bacterial patterns along narrow and broad elevational gradients, highlighting the profound potential impact of study scales and sampling schemes on observed elevation patterns. Second, this might be related to the different diversity indices, with some examining bacterial diversity at the phylum level, while some were the diversity of the whole community, including the number of OTU, Shannon and Simpson indices, which show different trends with increasing elevation^8,11,38^. Third, many factors, including abiotic (e.g. temperature, moisture, pH, and soil properties) and biotic factors (e.g. plant) changed along elevation gradients, and these factors would directly affect the bacterial community structure^3^, thus resulting in a variety of bacterial diversity distribution patterns. Therefore, the patterns of bacterial diversity in different mountain ecosystems were not consistent^4,25^. In the present study, there were significant differences in soil temperature, moisture, vegetation types, and soil properties with increasing elevation. All these factors might affect the bacterial community and even offset each other, leading to no difference in bacterial diversity. Also, compared with other studies^11,12,25,38^, the observed species, and Shannon index were relatively high at any elevations in this study. Under the condition of high bacterial diversity, it might not be easily affected by elevation.

Regardless of elevation, the soils were dominated by Proteobacteria and Acidobacteria (> 50%), but the relative abundance of Acidobacteria and other major taxa differed among the five elevations (Fig. 2; Table S3). Proteobacteria and Acidobacteria were the most abundant phylum in Wolong Nature Reserve, Western Sichuan Plateau^25^, in the Sygera Mountains of Tibet^13^, in Mount Gongga of the eastern Tibetan Plateau^36,39^, and they also predominate in other ecosystems, including subtropical bamboo plantations^16^ and temperate forest^38^. Therefore, the dominant bacterial phyla of different ecosystems and soil types are basically the same^38^. Proteobacteria were not different significantly among the five elevations, indicating that environmental differences caused by elevation had little effect on the dominant bacteria. Acidobacteria are known as oligotrophic and versatile heterotrophs characterized by low metabolic rates under low nutrient environments^36^, and their abundance is influenced by soil nutrition content and soil pH^13,39^. Yin et al.^40^ have found that Proteobacteria are more stable and seem not to respond to soil treatment compared with Acidobacteria, which was consistent with the present results.

Soil properties (e.g. soil pH and soil nutrient contents) and climate change can explain differences in the distribution of soil bacterial communities along elevational gradients^3^. Among these factors, pH and mean annual temperature were significantly correlated with bacterial community composition as well as the dominant bacterial phyla, classes, and genera^37^. Nottingham et al.^3^ have suggested that temperature plays an important role in shaping microbial community structures, which strongly affects soil bacterial diversity and composition^36^. Singh et al.^4^ have also found that climate, including the temperature and precipitation is the dominant driver of climate adaptation in bacterial niches. In this study, soil temperature has a crucial role in shaping the composition of bacterial communities (Fig. 3), both soil temperature and moisture were significantly correlated with the dominant bacterial phyla, except for Rokubacteria and Nitrospirae (Table S5). In addition, soil properties especially the soil pH and SBD also significantly influenced the bacterial phyla. The PLS-SEM in our study revealed that the direct effect of soil properties (pH, soil nutrients, and SBD) especially the SBD on bacterial community composition was stronger than that of soil temperature and moisture (Fig. 4). SBD can directly affect soil aeration and porosity, and it is an important factor affecting soil bacterial community^41,42^. Because most soil bacteria are aerobic, low SBD can provide sufficient oxygen for microorganisms and facilitate the nutrient circulation, thus contributing to the growth and development of soil bacteria^43^. Bhattacharya et al. also showed that soil bacterial alpha diversity and relative abundance of major phyla showed mid-elevation dip mainly caused by the direct impact of edaphic factors (soil organic carbon, total nitrogen, and moisture) but not temperature^44^, which is consistent with our results. Based on the above studies and the present results, differences of temperature and moisture caused by elevation were shown to significantly affect bacterial community composition, but the direct effect of temperature and moisture on soil bacterial community was limited under the long-term natural environmental condition. In order to further study of the direct impact of climate change on bacterial communities, soil translocation was conducted along the elevation gradient.

### Effects of soil translocation on soil bacterial community composition

Temperature changes caused by soil translocation would lead to changes in soil bacterial community composition, and the resulting composition in translocated soil is more similar to that in the new environment^20–22^. Soil microbial communities respond to temperature changes by acclimation, genotypic changes within a species (evolution), and species sorting^20^. Here, Proteobacteria and Acidobacteria were still dominant after soil translocation, which had no significant effect on their relative abundance (Fig. 5). Nottingham et al.^6^ have shown that most groups are not affected by temperature changes or were influenced by intrinsic soil properties. The present results showed that the two dominant phyla were relatively stable to climate change caused by soil translocation. Some other non-dominant bacterial phyla such as Rokubacteria, Gemmatimonadetes, Verrucomicrobia, and Latescibacteria, increased from higher to lower elevation, while the relative abundance of Bacteroidetes increased from lower to higher elevation, indicating that different species respond to climate change at different rates and directions, leading to an increase or decrease in the abundance of different groups. Consistent with the meta-analysis by Jiao et al. who found that soil abundant taxa persisted under the disturbances whereas rare taxa were more easily affected^45^, our results also suggest the higher resilience or resistance of soil dominant bacteria to environmental changes than that of non-dominant ones. The reason may be that dominant species effectively adapt to changed conditions by occupying a wide variety of niches and utilizing an array of resources, while rare species are strongly influenced by demographic stochasticity due to their small population sizes. Besides, the rare biosphere possessed a substantial amount of metabolically active lineages, thus presumably responding rapidly to local disturbances^45^. It was interesting that more groups showed significant changes for soil translated from H2200 to H1800 and H2600 (Fig. 6). Because both temperature and moisture showed direct positive effects on soil bacterial community structure (Fig. 4), the temperature and moisture of H2200 may be more suitable for soil bacteria than those of H1600 (with relatively high temperature and low moisture) and H2600 (with relatively low temperature and high moisture). Therefore, when soil was translated from H2200 to H1800 and H2600, the temperature and moisture become relatively unsuitable, thus the soil bacterial communities are more likely to respond the changes. Additionally, the responses of these phyla to temperature and moisture changes by soil translocation were not completely consistent with correlations between bacterial phyla and soil temperature and moisture along the elevation gradient (Table S5), suggesting that soil bacterial communities respond to the temperature change caused by short-term translocation might be different from long-term adaptability to temperature and moisture. Long-term temperature and moisture changes mainly affect the soil bacterial community by affecting the soil physical and chemical properties as discussed above, while short-term temperature and moisture interference has a certain direct impact on the non-dominant groups of the bacterial community.

Although some taxa changed when translocated, soil translocation did not significantly affect overall bacterial community structures. On the one hand, this might have been due to great influence by the dominant species as the two dominant phyla (Proteobacteria and Acidobacteria) did not change in this study. On the other hand, the bacterial community structure might have been influenced by the original elevation rather than the climate change caused by short-term reciprocal translocation. Yang et al.^19^ have also shown that the original elevation rather than short-term reciprocal translocation plays a greater role in determining the formation of microbial community. Similar to the present results, Puissant et al.^23^ have also found that a 4-year soil translocation did not affect bacterial community structure in subalpine grassland soils, and even 17 years after translocation, the dominant bacterial membership had not changed^24^. Therefore, the bacterial community appears to be resistant to changes in temperature and moisture or requires a longer time to exhibit a response^5^, which needs to further investigation.

### Metabolic function prediction of soil bacterial community

The present results showed that the function of metabolism had the highest relative abundance in all five elevations, although significant differences existed among different elevations. Metabolism was the primary function of soil microbial communities, which was consistent with other studies^46^. In secondary metabolic pathways, amino acid metabolism, carbohydrate metabolism, and metabolism of cofactors and vitamins were the main pathways, and the relative abundance of carbohydrate metabolism increased significantly with the increased elevation (Table 2), indicating that the metabolic potential of soil bacteria to carbohydrates increased at higher elevations. This result was consistent with the report that soil microorganisms in higher elevation areas prefer to use carbon source substrates of carbohydrate type^47^.

Although soil translocation changed some secondary metabolic pathways, the ratio of abundance in situ and in translocated soil was in the range of 0.92–1.09 (Fig. 7). The change in metabolic function was smaller than that of community composition (0.32–3.00), indicating that the function was more stable than community composition. Other studies have also found higher functional stability of soil microbial communities, such that microbial function is not affected even though there are changes in composition^48,49^. In addition, the functional redundancy of microorganisms could explain the stability of soil microbial functions^50,51^. Therefore, even though soil bacterial community composition changed, their metabolic function maintained relatively stable.

## Conclusions

In conclusion, the present results revealed that the bacterial community alpha diversity was not significantly different among the five elevations along 1600–3000 m at a mountainside in northwest Sichuan, but the bacterial community composition showed significantly different at phylum level. The changes of soil temperature and moisture along the elevation gradient mainly affected the soil bacterial community by affecting SBD, soil nutrients and pH. In response to reciprocal soil translocation for 12 months, some non-dominant bacterial phyla were directly affected by the changes of temperature and moisture caused by soil translocation, and the changes of soil bacterial metabolic functions were smaller than the changes of bacterial community composition. Our findings revealed that the soil dominant phyla and the soil bacterial metabolic functions may be relatively stable, and it is useful for understanding the ecosystem functional stability under climate change in this area.

### Supplementary Information

Supplementary Information.

## Data Availability

All data included in this study are available upon request by contact with the corresponding author.
