# Supplementary material for: Elevational characteristics of soil bacterial community and their responses to soil translocation at a mountainside in northwest Sichuan, China
Source: Sci Rep. 2023 Oct 20;13:17906. doi: 10.1038/s41598-023-44811-2 (PMC10589279; doi:10.1038/s41598-023-44811-2)
Supplement: Supplementary file 1 — Supplementary Information. [file 41598_2023_44811_MOESM1_ESM.docx]

Fig. S1 Rarefaction curves for soil bacteria of each soil sample in five elevations.


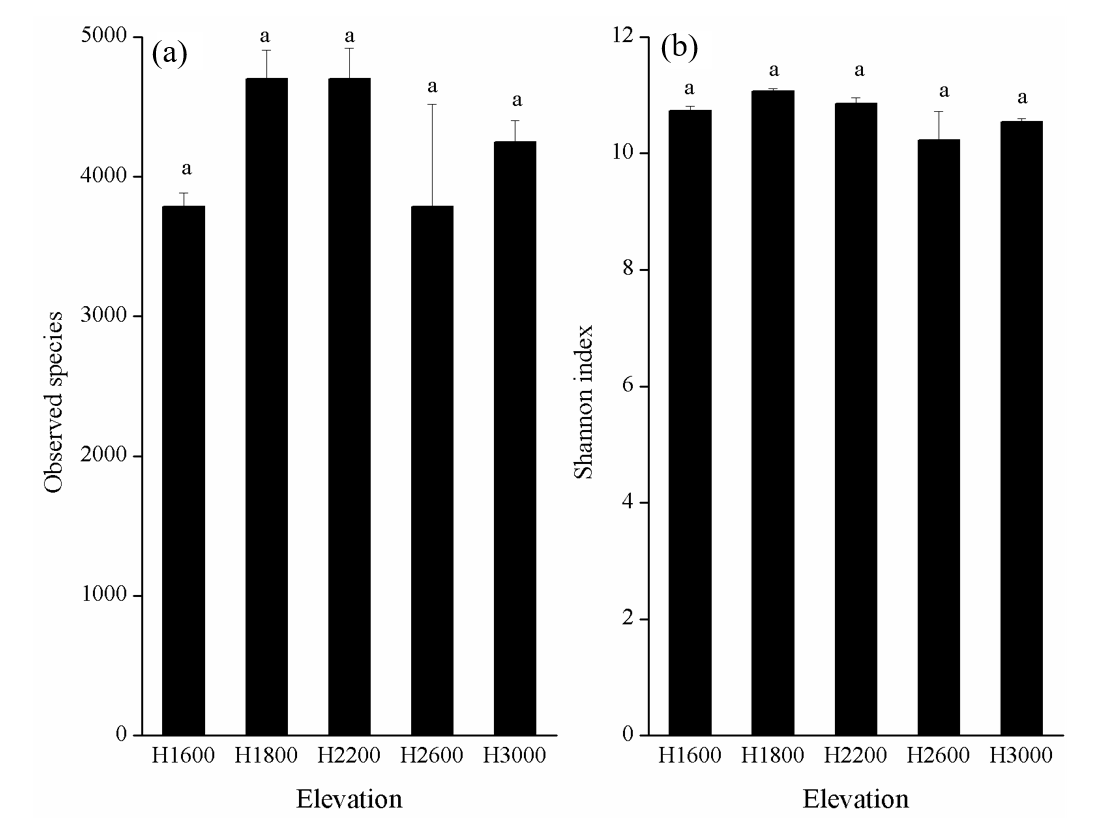


Fig.S2 OTUs and Shannon index for bacterial communities in five elevations


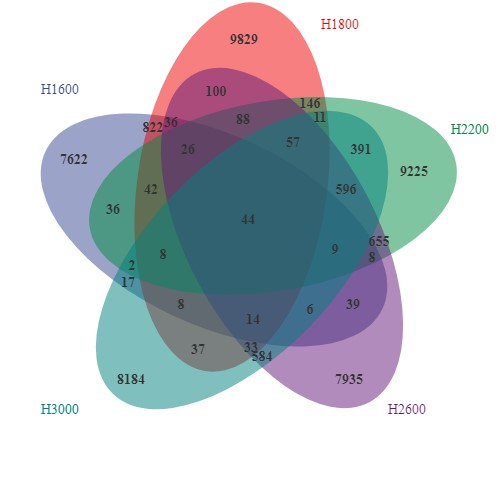


Fig. S3 Venn diagrams comparing the OTU memberships of the five elevations. The numbers in the figure represent the number of OTUs.

Table S1 Soil physical and chemical properties of soil sampling sites in five elevations.

| Soil properties | H1600 | H1800 | H2200 | H2600 | H3000 |
| --- | --- | --- | --- | --- | --- |
| SBD (g∙cm^-3^) | 1.35 ± 0.02 a | 1.36 ± 0.01 a | 0.75 ± 0.07 b | 1.05 ± 0.17 ab | 0.88 ± 0.01 b |
| pH | 5.51 ± 0.06 a | 5.41 ± 0.04 ab | 5.27 ± 0.07 ab | 5.08 ± 0.08 b | 5.05 ± 0.12 b |
| SOM (g∙kg^-1^) | 39.39 ± 26.08 a | 74.19 ± 6.93 a | 55.05 ± 5.68 a | 31.99 ± 5.90 a | 28.81 ± 5.54 a |
| TN (g∙kg^-1^) | 0.78 ± 0.23 a | 1.07 ± 0.15 a | 0.95 ± 0.07 a | 0.86 ± 0.08 a | 0.82 ± 0.04 a |
| TP (g∙kg^-1^) | 0.95 ± 0.18 a | 1.33 ± 0.06 a | 1.18 ± 0.07 a | 1.40 ± 0.07 a | 1.00 ± 0.04 a |
| TK (g∙kg^-1^) | 17.70 ± 2.65 a | 16.30 ± 1.12 a | 17.63 ± 0.39 a | 21.95 ± 0.13 a | 20.00 ± 1.38 a |
| AN (mg∙kg^-1^) | 127.64 ± 28.73 ab | 220.45 ± 33.79 a | 139.28 ± 6.51 ab | 127.28 ± 9.71 ab | 108.23 ± 14.64 a |
| AP (mg∙kg^-1^) | 10.79 ± 2.16 a | 12.14 ± 2.63 a | 17.49 ± 0.92 a | 20.51 ± 0.80 a | 14.32 ± 3.32 a |
| AK (mg∙kg^-1^) | 51.55 ± 13.71 a | 54.98 ± 17.02 a | 132.81 ± 42.38 a | 87.51 ± 19.81 a | 79.43 ± 20.46 a |

SBD: soil bulk density, SOM: soil organic matter, TN: total nitrogen, TP: total phosphorus, TK: total potassium, AN: available nitrogen, AP: available phosphorus, AK: available potassium. Different lowercase letters indicate significant difference among different elevations (*P* < 0.05).

Table S2 Correlation coefficients between soil bacterial alpha diversity indices and environmental factors

| Environmental factors | Observed species | Shannon |
| --- | --- | --- |
| Temperature | 0.034 | 0.393 |
| Moisture | 0.049 | -0.315 |
| SBD | 0.127 | 0.469 |
| pH | 0.026 | 0.34 |
| SOM | 0.388 | 0.509 |
| TN | 0.464 | 0.386 |
| TP | 0.101 | 0.004 |
| TK | -0.304 | -0.499 |
| AN | 0.334 | 0.456 |
| AP | -0.011 | -0.292 |
| AK | 0.16 | -0.171 |

Table S3 Bacterial phyla composition and relative abundance in soils under five elevations.

| Bacterial phyla | H1600 | H1800 | H2200 | H2600 | H3000 |
| --- | --- | --- | --- | --- | --- |
| Proteobacteria | 36.67 ± 1.39 a | 41.79 ± 0.42 a | 34.95 ± 1.68 a | 32.95 ± 3.69 a | 33.18 ± 0.72 a |
| Acidobacteria | 21.12 ± 1.24 b | 25.13 ± 0.24 ab | 30.15 ± 1.58 a | 27.22 ± 2.60 ab | 26.30 ± 0.01 ab |
| Chloroflexi | 6.59 ± 0.93 c | 6.31 ± 0.70 c | 5.70 ± 0.48 c | 10.87 ± 1.57 b | 15.10 ± 0.05 a |
| Rokubacteria | 7.88 ± 1.39 ab | 3.02 ± 0.47 b | 9.67 ± 0.55 a | 10.74 ± 2.06 a | 5.47 ± 0.19 ab |
| Actinobacteria | 6.25 ± 0.11 ab | 7.13 ± 0.63 a | 3.28 ± 0.28 b | 4.29 ± 1.38 ab | 4.43 ± 0.22 ab |
| Gemmatimonadetes | 5.48 ± 0.32 a | 3.10 ± 0.30 b | 2.60 ± 0.43 b | 2.35 ± 0.09 b | 1.91 ± 0.07 b |
| Bacteroidetes | 2.43 ± 0.44 b | 6.00 ± 0.55 a | 1.72 ± 0.20 b | 1.69 ± 0.49 b | 1.64 ± 0.24 b |
| Verrucomicrobia | 0.53 ± 0.20 c | 1.38 ± 0.37 c | 3.62 ± 0.30 ab | 2.29 ± 0.85 bc | 4.65 ± 0.19 a |
| Nitrospirae | 3.15 ± 0.37 a | 1.09 ± 0.25 b | 1.61 ± 0.34 b | 2.19 ± 0.09 ab | 1.92 ± 0.06 b |
| Latescibacteria | 3.98 ± 0.18 a | 1.00 ± 0.06 bc | 1.43 ± 0.09 b | 0.80 ± 0.19 c | 0.69 ± 0.02 c |

Different lowercase letters indicate significant difference among different elevations (*P* < 0.05).

Table S4 Fitting results of environmental factors in NMDS analysis

| Environmental factors | NMDS1 | NMDS2 | r^2^ | *P* |
| --- | --- | --- | --- | --- |
| Temperature | -0.658 | -0.753 | 0.781 | 0.001 |
| Moisture | 0.888 | 0.460 | 0.597 | 0.006 |
| SBD | -0.980 | -0.198 | 0.670 | 0.001 |
| pH | -0.593 | -0.805 | 0.476 | 0.018 |
| SOM | -0.850 | 0.527 | 0.360 | 0.072 |
| TN | -0.572 | 0.820 | 0.201 | 0.265 |
| TP | -0.208 | 0.978 | 0.087 | 0.594 |
| TK | 0.984 | -0.181 | 0.305 | 0.108 |
| AN | -0.817 | 0.577 | 0.407 | 0.035 |
| AP | 0.936 | 0.351 | 0.238 | 0.199 |
| AK | 0.909 | -0.416 | 0.255 | 0.182 |

Table S5 Correlation coefficients between environmental factors and the relative abundances of the top 10 bacterial phyla

| Bacterial phyla | Temperature | Moisture | SBD | pH | SOM | TN | TP | TK | AN | AP | AK |
| --- | --- | --- | --- | --- | --- | --- | --- | --- | --- | --- | --- |
| Proteobacteria | 0.568* | -0.652** | 0.656** | 0.339 | 0.665** | 0.453 | 0.257 | -0.539* | 0.686** | -0.273 | -0.349 |
| Acidobacteria | -0.441 | 0.636* | -0.781** | -0.411 | 0.087 | 0.177 | 0.337 | 0.028 | -0.039 | 0.477 | 0.625* |
| Chloroflexi | -0.779** | 0.482 | -0.378 | -0.547* | -0.602* | -0.363 | -0.269 | 0.533* | -0.545* | 0.112 | -0.063 |
| Rokubacteria | -0.169 | 0.509 | -0.471 | -0.096 | -0.507 | -0.415 | -0.057 | 0.488 | -0.519* | 0.380 | 0.366 |
| Actinobacteria | 0.582* | -0.820*** | 0.845*** | 0.389 | 0.347 | 0.163 | 0.128 | -0.304 | 0.450 | -0.534* | -0.572* |
| Gemmatimonadetes | 0.721** | -0.707** | 0.566* | 0.648** | 0.116 | -0.080 | -0.224 | -0.362 | 0.067 | -0.475 | -0.244 |
| Bacteroidetes | 0.579* | -0.649** | 0.619* | 0.410 | 0.681** | 0.543* | 0.311 | -0.580* | 0.787** | -0.301 | -0.292 |
| Verrucomicrobia | -0.809*** | 0.730** | -0.632* | -0.687** | -0.199 | 0.057 | -0.070 | 0.293 | -0.267 | 0.411 | 0.423 |
| Nitrospirae | 0.153 | -0.126 | 0.205 | 0.229 | -0.512 | -0.604* | -0.478 | 0.340 | -0.480 | -0.227 | -0.377 |
| Latescibacteria | 0.618* | -0.528* | 0.480 | 0.624* | 0.028 | -0.145 | -0.412 | -0.314 | -0.094 | -0.393 | -0.268 |

* *P* < 0.05; ** *P* < 0.01; *** *P* < 0.001.

Table S6 Standardized effects of soil bacterial community and its affecting factors

| Dependent | Independent | Direct effects | Indirect effects | Total effects |
| --- | --- | --- | --- | --- |
| Elevations | Moisture | 0.821 | 0.000 | 0.821 |
|  | Temperature | -0.954 | 0.000 | -0.954 |
| Moisture | pH | -0.149 | 0.000 | -0.149 |
|  | SBD | -0.410 | 0.000 | -0.410 |
|  | Nutrients | 0.158 | 0.000 | 0.158 |
|  | Community | 0.280 | 0.193 | 0.473 |
| Temperature | pH | 0.673 | 0.000 | 0.673 |
|  | SBD | 0.406 | 0.000 | 0.406 |
|  | Nutrients | 0.483 | 0.000 | 0.483 |
|  | Community | 0.071 | -0.386 | -0.315 |
| pH | Community | -0.004 | 0.000 | -0.004 |
| SBD | Community | -0.585 | 0.000 | -0.585 |
| Nutrients | Community | -0.302 | 0.000 | -0.302 |
